# The Transient Nature of Bunyamwera Orthobunyavirus NSs Protein Expression: Effects of Increased Stability of NSs Protein on Virus Replication

**DOI:** 10.1371/journal.pone.0064137

**Published:** 2013-05-08

**Authors:** Ingeborg van Knippenberg, Rennos Fragkoudis, Richard M. Elliott

**Affiliations:** 1 Biomedical Sciences Research Centre, University of St. Andrews, St. Andrews, Fife, Scotland, United Kingdom; 2 The Roslin Institute and Centre for Infectious Diseases, University of Edinburgh, Edinburgh, Scotland, United Kingdom; Kantonal Hospital St. Gallen, Switzerland

## Abstract

The NSs proteins of bunyaviruses are the viral interferon antagonists, counteracting the host's antiviral response to infection. During high-multiplicity infection of cultured mammalian cells with Bunyamwera orthobunyavirus (BUNV), NSs is rapidly degraded after reaching peak levels of expression at 12hpi. Through the use of inhibitors this was shown to be the result of proteasomal degradation. A recombinant virus (rBUN4KR), in which all four lysine residues in NSs were replaced by arginine residues, expresses an NSs protein (NSs4KR) that is resistant to degradation, confirming that degradation is lysine-dependent. However, despite repeated attempts, no direct ubiquitylation of NSs in infected cells could be demonstrated. This suggests that degradation of NSs, although lysine-dependent, may be achieved through an indirect mechanism. Infection of cultured mammalian cells or mice indicated no disadvantage for the virus in having a non-degradable NSs protein: in fact rBUN4KR had a slight growth advantage over wtBUNV in interferon-competent cells, presumably due to the increased and prolonged presence of NSs. In cultured mosquito cells there was no difference in growth between wild-type BUNV and rBUN4KR, but surprisingly NSs4KR was not stabilised compared to the wild-type NSs protein.

## Introduction

Bunyamwera virus (BUNV) is the type species of both the family *Bunyaviridae* and the genus *Orthobunyavirus*. This family contains pathogens of serious concern such as Rift Valley fever virus and Crimean-Congo haemorrhagic fever virus. BUNV itself is of low pathogenicity ([1]), but the recent outbreak of Schmallenberg orthobunyavirus in Europe ([2]) underscores the pathogenic potential of the orthobunyaviruses and the economic damage these viruses can cause. All members of the *Bunyaviridae* except those in the *Hantavirus* genus are transmitted by arthropods. Bunyaviruses possess a trisegmented RNA genome of negative or partially ambisense polarity that is encapsidated by nucleoprotein (N) and bound to the viral RNA polymerase (L), and is enveloped in a host-derived membrane containing the viral glycoproteins. Orthobunyaviruses encode the viral RNA polymerase on the large (L) genome segment, a polyprotein precursor on the medium (M) segment, and the N protein and a nonstructural protein (NSs), in overlapping reading frames, on the S segment. The M segment-encoded polyprotein is co-translationally cleaved to yield the mature glycoproteins Gn and Gc as well as a second nonstructural protein, NSm ([3]). The NSs protein is the viral interferon (IFN) antagonist ([4], [5], [6]) but has also been implicated in other functions such as regulation of translation, apoptosis, and viral polymerase activity ([7], [8]–[11]).

The observation that the levels of NSs protein decline rapidly after 12 hours post infection (hpi), whereas the rate of *de novo* synthesis remains unchanged during this time ([8], [12], [13]), suggested that NSs is subject to active targeted proteasomal degradation. Proteins are targeted for proteasomal degradation by covalent attachment of multiple ubiquitin molecules (ubiquitylation) at lysine (K) residues. The three-step ubiquitylation reaction involves the E1 activating enzyme, an E2 conjugating enzyme and an E3 ligase complex. The E3 ligases are the components that confer specificity on this system by recognising the target proteins ([14]). The ubiquitin-proteasome system (UPS) is involved in host anti-viral defenses by regulating the degradation or activation of crucial factors, and viruses have evolved mechanisms either to block these signals or to subvert the UPS to cause degradation of anti-viral factors ([15]–[20]). In other cases, viruses have been found to require a functional UPS for specific steps in their replication cycle such as entry ([21], [22]), nuclear export ([23]), budding ([24]), genome transcription/translation/replication ([25]–[27]) or general virus replication ([28]–[33]). Given these extensive interactions of viruses from diverse families with the UPS some involvement of this system in the BUNV replication cycle could also be expected.

We present here an analysis of the degradation of NSs and the effect of mutations that prevent this degradation. Both the use of proteasome inhibitors and mutation of the lysine residues in NSs to arginine residues resulted in accumulation of NSs, suggesting that NSs is targeted for proteasomal degradation through ubiquitylation. Interestingly, a recombinant virus that expresses an NSs protein lacking lysine residues behaved almost indistinguishably from wild-type (wt) virus in infection of both cultured mammalian cells and mice, displaying only a very slight growth advantage in interferon-competent A549 cells. Surprisingly, in mosquito cells the mutant NSs4KR protein was even less stable than wt NSs, suggesting the involvement of an alternative degradation mechanism.

## Results

### Degradation of NSs is proteasome-mediated and lysine-dependent

**Figure 1:**
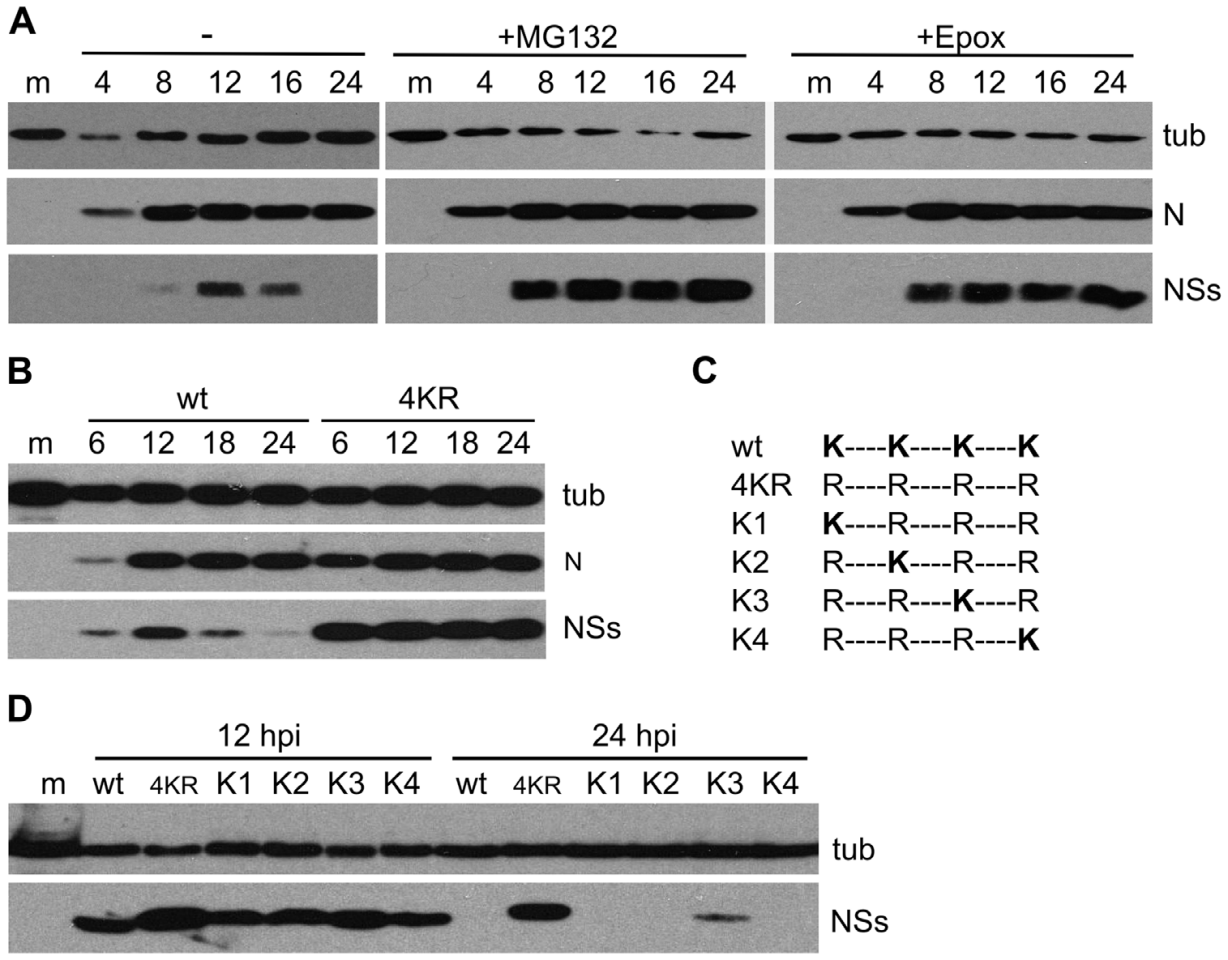
Degradation of NSs. BHK cells were infected at 5 PFU/cell and harvested at the indicated times post infection (top of blots) and cell lysates were analysed by immunoblotting using antibodies against the proteins indicated to the right of the panels (tub  =  tubulin). (A) Effect of proteasome inhibitors. Cells were mock-infected or infected with wtBUNV and either left untreated or treated with MG132 (10 μM) or Epoxomicin (200 nM) from 5 hpi until harvest. (B) Expression of 4KRNSs. Cells were mock-infected or infected with wtBUNV or rBUN4KR. (C) Schematic overview of the NSs proteins of recombinant viruses expressing NSs proteins containing a single lysine at one of the four positions (K1-K4). (D) Expression of NSs containing single lysine residues. Cells were mock-infected or infected with wtBUNV, rBUN4KR, or rBUN-K1, -K2, -K3 and -K4. m =  mock-infected; wt  =  wtBUNV; 4KR  =  rBUN4KR.

During high-multiplicity infection of BHK cells with wtBUNV, NSs protein levels increased up to 12hpi and then decreased to below detection limits by 24hpi, whereas N protein levels reached a plateau from approximately 12hpi (Fig. 1 A, left panel). Since N and NSs are translated from overlapping ORFs on the same mRNA, this suggested that NSs was actively degraded during infection. Treating infected cells with the proteasome inhibitor MG132 prevented degradation and led to considerable accumulation of NSs (Fig. 1A, middle panel). MG132 also blocks lysosomal proteases and therefore infected cells were treated with the more specific proteasome inhibitor epoxomicin (Fig. 1A, right panel). This resulted in accumulation of NSs to a similar extent as with MG132 treatment, confirming that NSs is indeed subject to proteasomal degradation.

Proteasomal protein degradation is regulated through ubiquitylation of the substrate at lysine (K) residues. BUNV NSs has 4 lysine residues, at amino acid (aa) positions 39, 44, 49 and 54. Although these residues are not conserved in all the NSs proteins encoded by viruses in the *Orthobunyavirus* genus, the lysine residue at position 44 is completely conserved in viruses of the Bunyamwera serogroup. Using reverse genetics we generated a virus in which all four lysine residues were mutated to arginine (R), without affecting the sequence of the overlapping N protein. The mutant virus was called rBUN4KR and the mutant protein NSs4KR.

In BHK cells infected with the mutant virus, the NSs4KR protein was greatly stabilised (Fig. 1B), reaching levels similar to those seen for wt NSs in wtBUNV infected cells treated with MG132 (compare Fig. 1A). The increased stability of NSs4KR suggested that modification of wt NSs at one or more of the lysine residues, most likely by ubiquitylation, is involved in its degradation. Four additional recombinant mutant viruses were created in which the four arginine residues in rBUN4KR were individually mutated back to lysine (called rBUN-K1 to K4; Fig. 1C). When BHK cells were infected with these viruses, all the single-lysine NSs proteins were subject to degradation (Fig. 1D). The NSs protein containing a lysine residue at position 49 (rBUN-K3) was only partially degraded after 24 hours, indicative of slower processing. This would agree with structure predictions (Jpred, http://www.compbio.dundee.ac.uk/www-jpred), that place this lysine residue in a less solvent-accessible position in the protein and thus less available for modification (Rob Hagan, personal communication). Taken together these results strongly suggest that NSs is subject to proteasomal degradation and that any of the four lysine residues could be used to target NSs to the proteasome.

### Ubiquitylation of NSs

**Figure 2:**
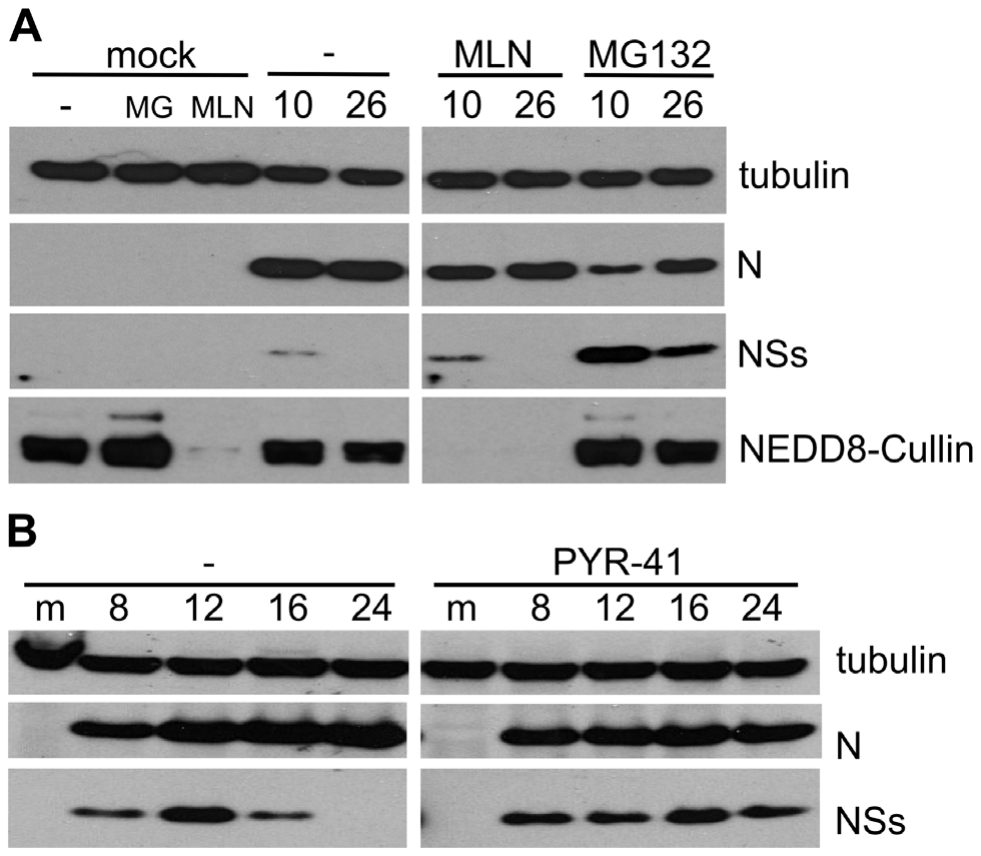
Effect of ubiquitin ligase E3 and E1 inhibitors on NSs degradation. Cells were infected at 5 PFU/cell and harvested at the indicated times post infection (top of blots) and cell lysates were analysed by immunoblotting using antibodies against the proteins indicated to the right of the panels. (A) Effect of E3 inhibitor. A549 cells were mock-infected or infected with wtBUNV and left untreated (−) or treated with MG132 (10 μM; MG) or MLN4924 (1 µM; MLN) from 5 hpi until harvest. (B) Effect of E1 inhibitor. BHK cells were mock-infected (m) or infected with wtBUNV and left untreated (−) or treated with PYR-41 (50 µM) from 8 hpi.

Med8 is part of the mediator complex that regulates cellular RNA polymerase II (RNAPII) activity, and in addition can form a Cullin-RING E3 ubiquitin ligase complex together with Elongins B and C (EloB, EloC), Cullin2 (Cul2) and RING-box protein 1 (Rbx1) ([34]). Med8 has previously been reported to interact with BUNV NSs ([35]), and thus Med8 was the prime candidate for the E3 ligase that targets NSs for degradation. Cullin-RING ligases require activation through neddylation to form active E3 ligase complexes ([36]). This activation is accomplished by NEDD8-activating enzyme (NAE) which can be blocked by using the inhibitor MLN4924 ([37]). A549 cells treated with this inhibitor indeed showed greatly reduced levels of neddylated Cullins (Fig. 2A). However, in contrast to the effects of MG132, treating BUNV-infected cells with MLN4924 did not stabilise NSs (Fig. 2A), indicating that NSs degradation is not regulated through Cullin-RING E3 ubiquitin ligases. This excluded the possibility that NSs degradation is mediated by the Med8-containing E3 ligase.

To elucidate further the mechanism of NSs degradation, infected BHK cells were treated with PYR-41, an inhibitor of ubiquitin activating enzyme E1 (UAEΙ; ([38]); Fig. 2B). If ubiquitylation was required for NSs degradation, blocking E1 should have a stabilising effect on NSs. Indeed, PYR-41 treatment did result in stabilisation of NSs (Fig. 2B), although apparently to a lower extent than observed with MG132 (compare Fig. 1A). On closer examination the drug appeared to have an overall inhibitory effect on viral protein expression. When PYR-41 was added an hour prior to or just after infection of the cells, no or only very little N protein could be detected by 24hpi (not shown), confirming a general inhibitory effect of the drug on BUNV replication. Thus, the drug seemed to inhibit viral protein synthesis in addition to blocking degradation of NSs, indicating that NSs degradation does indeed require ubiquitylation.

Since all the evidence presented above pointed towards a classical ubiquitylation-proteasomal degradation pathway for NSs, efforts were made to demonstrate directly the existence of ubiquitylated NSs protein. *In vitro* ubiquitylation assays ([17]) failed to show the generation of any higher molecular weight species of NSs (data not shown). *In vivo* ubiquitylation was investigated using an A549-derived cell line expressing 6×His-tagged ubiquitin, A549-6His-Ub. The cells were infected with wtBUNV and treated with MG132. The presence of ubiquitylated NSs was investigated by passing cell lysates over Ni^2+^-resin, but no bound NSs was detected (data not shown). NSs expression levels were estimated to be ∼25–50 times lower in A549 cells compared to BHK cells, which might result in the levels of ubiquitylated NSs being below the detection limits for this method. It should also be noted that no higher molecular weight species were detected by western blotting with our anti-NSs antibody of lysates of infected BHK, A549, or A549-6His-Ub cells, whether or not the cells were treated with inhibitors.

In summary, although degradation of NSs was found to be dependent on an active UPS, it was not possible to confirm ubiquitylation of NSs directly.

### Effect of the 4KR mutations on known functions of NSs

**Figure 3:**
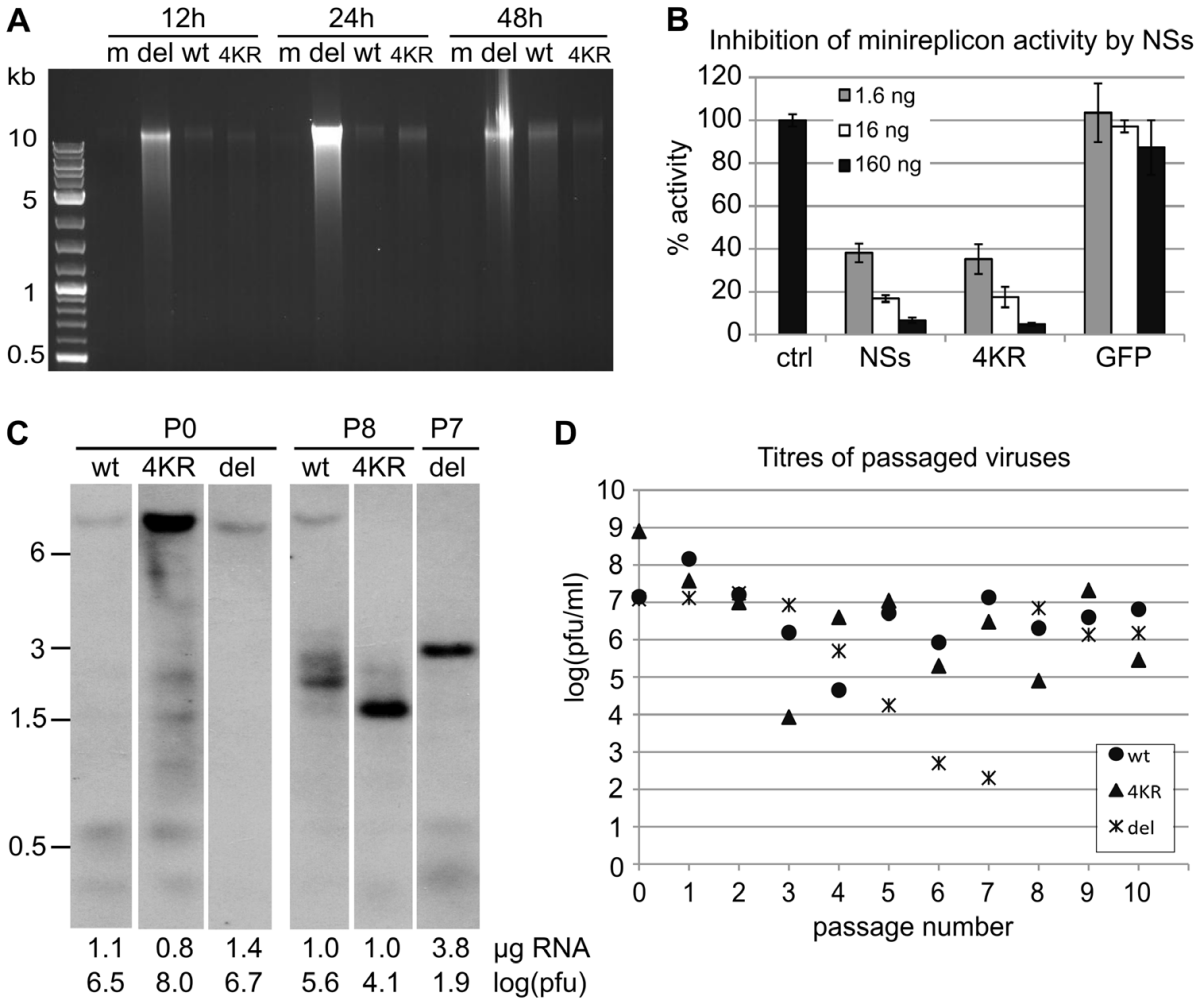
Effect of NSs4KR mutations on known functions of NSs. (A) Induction of apoptosis in infected cells as measured by DNA fragmentation. A549 cells were mock-infected or infected with wtBUNV, rBUN4KR, or rBUNdelNSs2. Cells were harvested at 12, 24, and 48 hpi and analysed for DNA fragmentation. (B) Regulation of the viral polymerase in a minireplicon assay. Increasing amounts of plasmids (1.6–160 ng) expressing wt NSs or NSs4KR or a nonrelated protein (GFP) were added to a minireplicon assay expressing the viral minigenome (expressing *Renilla* luciferase), viral polymerase and nucleoprotein. Values shown are from quadruplicate measurements and represent *Renilla* luciferase activity over firefly luciferase activity, normalised to the control reaction. (C) Generation of defective RNAs. wtBUNV, rBUNdelNSs2, and rBUN4KR viruses were serially passaged at a fixed volume in BHK cells. Virions from the virus stock (P0) and passage 8 (P8; or P7 for rBUNdelNSs2) were pelleted and their extracted RNA was analysed by Northern blotting using a probe to detect genome-sense L RNA. All lanes are from the same exposure of the same Northern blot. Positions of size markers (in kb) are indicated to the left, and the amount of RNA and PFU-equivalents (log(pfu)) loaded are indicated below the blot. (D) Titres of the viruses at each passage.

One of the functions of NSs is to block apoptosis, as a virus deficient in NSs expression, rBUNdelNSs2, was shown to be a potent inducer of programmed cell death ([9]). In order to study the effect of the mutant NSs4KR on induction of apoptosis, infected cells were subjected to DNA fragmentation analysis (Fig. 3A). Although all infected cells showed some degree of DNA laddering compared to the mock-infected cells, this was barely detectable in cells infected with wt BUNV or rBUN4KR, whereas rBUNdelNSs-infected cells showed a bright smear of fragmented DNA as early as 12hpi (Fig. 3A).

Another reported function of NSs is to regulate the viral RNA polymerase activity, as demonstrated by the inhibitory effect of co-expression of NSs in a minireplicon assay ([11]). Therefore, we examined whether the lysine-to-arginine mutations in NSs4KR affected its ability to control the viral polymerase. When NSs4KR was co-expressed in the minireplicon assay, it displayed the same dose-dependent inhibitory effect as co-expression of wt NSs protein (Fig. 3B), indicating that the lysine residues in NSs were not required for the polymerase-regulating function.

Regulation of the viral polymerase by NSs could be important for reducing the generation of defective interfering particles (DIs), which contain truncated copies of the viral genome segments ([12], [39]) and could act as strong IFN inducers ([40]). To investigate whether NSs stability was related to DI formation, wtBUNV, rBUN4KR and rBUNdelNSs2 were serially passaged in BHK cells, starting at 5 PFU/cell and using a fixed volume of the infectious supernatant thereafter. After 8 passages (P8; or P7 for rBUNdelNSs2) the RNA was extracted from pelleted virions and analysed by Northern blotting with an L segment-specific probe (Fig. 3C). All three viruses had acquired smaller L-segment-derived RNA species, characteristic of DI RNAs ([39]), indicating that increased and prolonged expression of NSs does not prevent DI formation during passage in BHK cells. Both the rBUN4KR and rBUNdelNSs2 virus had accumulated relatively larger amounts of DI RNAs than wtBUNV whereas the relative levels of full-length L segment of these viruses were below the detection limit in these experimental conditions. We define the PFU-equivalent as the number of PFUs used to extract a certain amount of RNA. The amount of full-length L segment would be expected to correlate with the amount of PFUs since these require a functional polymerase. Thus, for rBUN4KR P0 more PFU-equivalents were loaded onto the gel than for wtBUNV P0 (10^8^ v 10^6.5^) and the signal for full length L segment is correspondingly stronger and seems to be proportional to the PFU-equivalent. For the P8/P7 samples, due to volume constraints only relatively low PFU-equivalents were loaded and this seemed to correlate with a low signal for full-length L segment for wt BUNV and the absence of this signal for the rBUN4KR and rBUNdelNSs2 viruses.

The titres of infectious virus released into the medium varied considerably with the different passages (Fig. 3D). As the cells were inoculated with fixed volumes at each passage, the m.o.i. for each infection also varied markedly. Interestingly, despite the presence of large amounts of DI RNAs at P8/P7 all viruses grew to titres in the range 10^6^–10^7^ PFU/ml at P9/P8.

Viral RNA of passage 10 (P10) was extracted from pelleted virions and the S segments were amplified by RT-PCR followed by sequence analysis. The S segment of rBUN4KR had retained its K-to-R mutations and no mutations were found in the wtBUNV S segment. Interestingly, the S segment of rBUNdelNSs2 had acquired the GTG-to-ATG mutation at NSs codon 22 that had been observed previously ([13]).

### Stabilised NSs expression provides a growth advantage in IFN-competent mammalian cells

**Figure 4:**
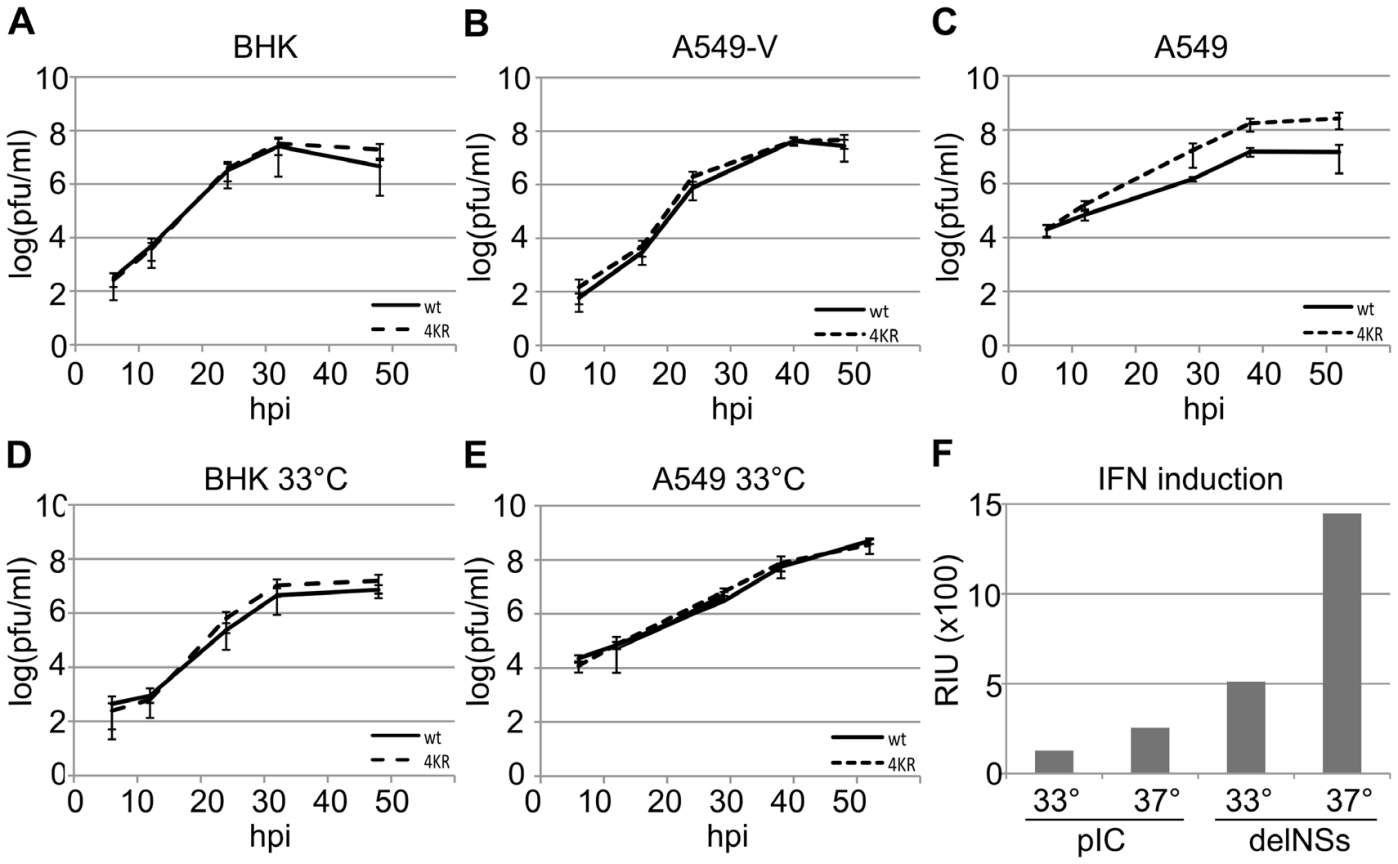
Virus growth and IFN induction in cultured mammalian cells. (A–E) Multicycle growth kinetics of wtBUNV and rBUN4KR were compared in BHK (A, D), A549 (C, E) and A549-V (B) cells by infecting triplicate wells at 0.01 PFU/cell and titrating the supernatants at the indicated time points. A–C: incubation at 37°C; D, E: incubation at 33°C. (F) Induction of IFN by treatment with poly(I∶C) or infection with rBUNdelNSs2. A549 cells were incubated at 33°C or 37°C and the supernatants assayed after 24 h. The amount of IFN produced is expressed as relative IFN units (RIU), defined as RIU  = 2^N^ where N =  the number of two-fold dilutions of the supernatants that protected the reporter cells from EMCV challenge (see methods).

In order to study the effect of stabilisation of NSs during virus replication, the growth kinetics of wtBUNV and rBUN4KR were compared in IFN-competent (A549) and IFN-incompetent (BHK and A549-V) cells. In both BHK and A549-V cells (Fig. 4A and B, respectively), rBUN4KR behaved almost indistinguishably from wt BUNV. In naïve A549 cells, however, the mutant virus reached titers of 2.7×10^8^ PFU/ml whereas wt virus grew to 2.8×10^7^ PFU/ml (Fig. 4C). This difference suggests that the increased and prolonged presence of NSs in rBUN4KR conferred a growth advantage in IFN competent cells. Interestingly, this growth advantage was not observed when the infections were incubated at 33°C (Fig. 4E). At this temperature, rBUN4KR and wtBUNV displayed similar growth characteristics in both BHK and A549 cells (Fig. 4D and E, respectively).

To understand why this difference in growth was observed only at 37°C, we examined IFN production in A549 cells at both temperatures. Cells were either treated with poly(I∶C) or infected with rBUNdelNSs2, a potent IFN inducer ([6]), for 24 h. In a protection assay on A549-NPro cells, the relative IFN content of the supernatants from the treated or infected cells was measured by monitoring the number of 2-fold dilutions of supernatant that protected the cells from subsequent infection by IFN-sensitive EMCV. Both poly(I∶C) treatment and rBUNdelNSs infection induced about twice as much IFN at 37°C as at 33°C, but virus infection appeared to be a much stronger inducer than the dsRNA analogue (Fig. 4F). When cells were infected with either wtBUNV or rBUN4KR, no IFN could be detected in the supernatant (not shown), indicating that both viruses were successful in blocking the IFN response. Thus, while wtBUNV was capable of overcoming the IFN response at either temperature, this appeared to come at a slight fitness cost at 37°C, whereas rBUN4KR grew with equal efficiency at both temperatures.

### Virulence in mice is not significantly altered with rBUN4KR virus

**Figure 5:**
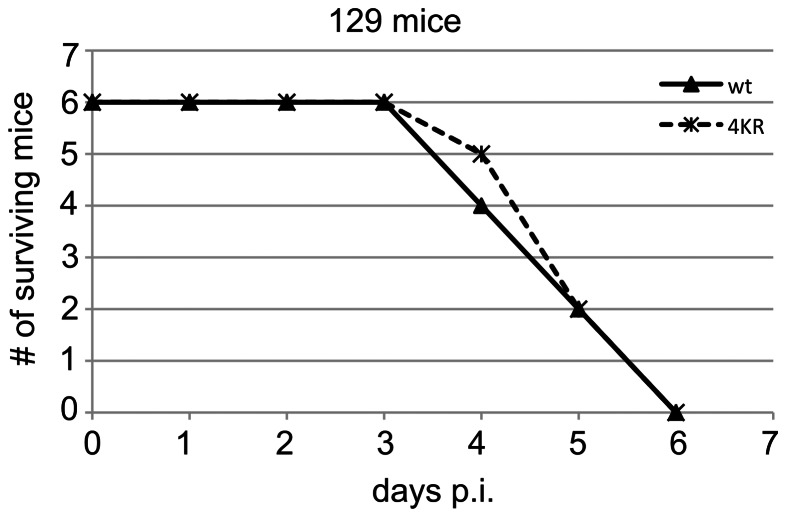
Virulence in mice. Assessment of wtBUNV and rBUN4KR virulence in mice. Strain 129 mice were infected i.c. with 1000 PFU of wtBUNV or rBUN4KR virus. The mice were monitored twice daily and sacrificed when moribund.

Since the mutant rBUN4KR virus appeared more capable to counteract the IFN response in A549 cells, we examined whether the 4KR mutations also led to increased virulence in mice. To this end, 129-strain mice were infected intracranially (i.c) with wtBUNV or rBUN4KR (Fig. 5) and monitored for up to two weeks. Similar to previous observations ([4], [41]), wtBUNV-infected mice died at 4–6 dpi. The rBUN4KR-infected mice died at a similar rate to wtBUNV-infected mice, demonstrating that expression of the stabilised NSs protein did not alter the virulence of the virus in this mouse model.

### Virus replication in mosquito cells

**Figure 6:**
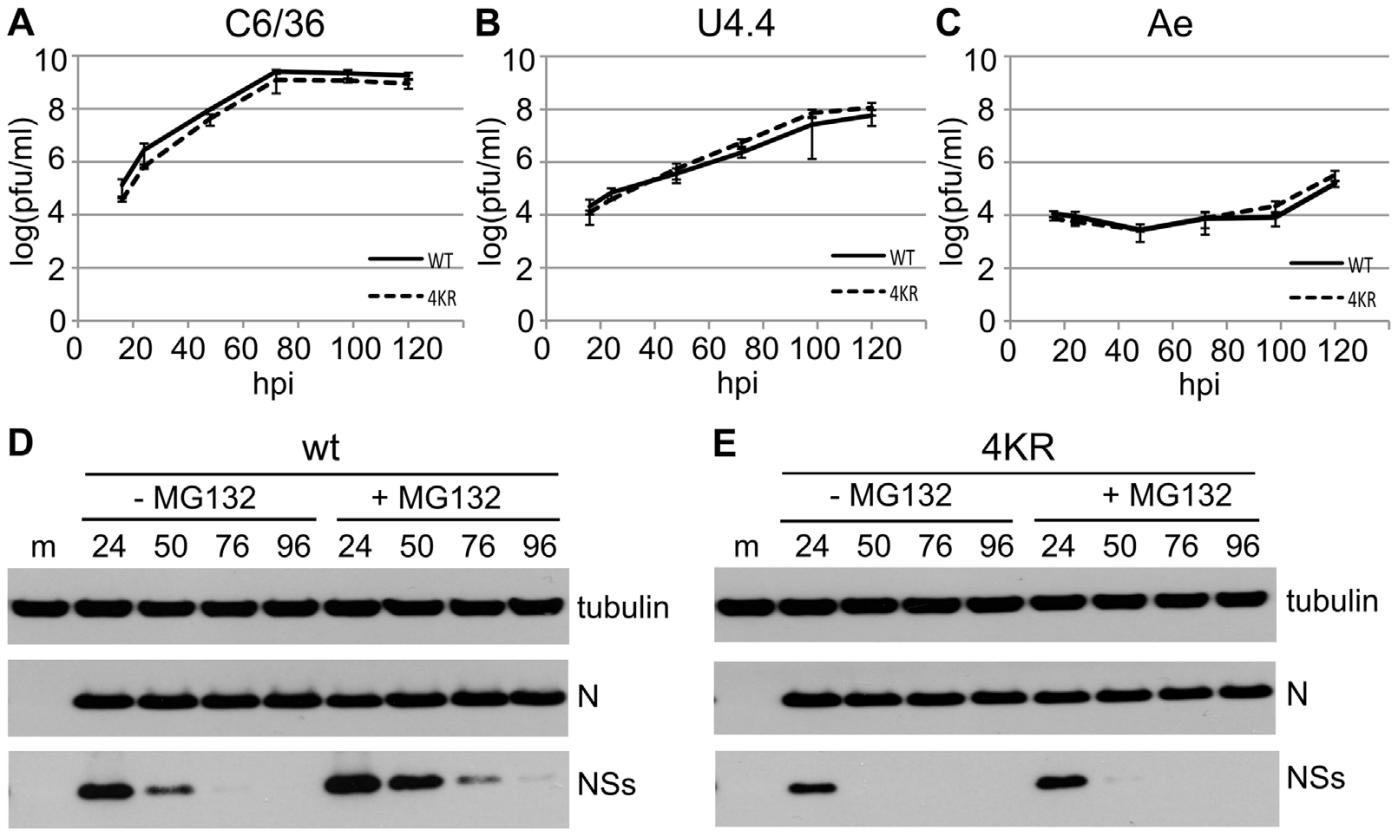
wtBUNV and rBUN4KR infection of mosquito cells. (A–C) Virus growth in mosquito cells. Triplicate wells of C6/36, U4.4, or Ae cells were infected at 0.01 PFU/cell and supernatants analysed by plaque assay titration. (D, E) Expression of NSs protein. C6/36 cells were infected with wtBUNV (D) or rBUN4KR (E) at 5 PFU/cell, and left untreated or treated with MG132 for 8 h prior to harvesting. Cell lysates were analysed by immunoblotting using antibodies against the proteins indicated to the right. Above the lanes, m  =  mock; numbers are hpi.

Since the mutations in rBUN4KR that prevented degradation of NSs did not seem to affect replication of the virus in a negative way either in mammalian cells or in mice, we investigated whether the lysine residues in NSs may be important for some aspect of the virus replication cycle in mosquito cells. *Aedes albopictus*-derived C6/36 and U4.4 cells, and *Aedes aegypti*-derived Ae cells were infected with wtBUNV or rBUN4KR at 0.01 PFU/cell and the titres of released virus were measured over time (Fig. 6A–C). The growth kinetics of both viruses were similar in the same cells, but the viruses replicated more efficiently in the *A. albopictus* cells than in the *A. aegypti* cells.

Viral protein expression was analysed in C6/36 cells infected with 5 PFU/cell (Fig. 6D, E). Both viruses produced easily detectable levels of N protein by 24 hpi. The rate of synthesis of NSs as compared to N protein during wtBUNV infection had previously been shown to be significantly lower in C6/36 cells than in BHK cells ([12]). Accordingly, detection of NSs protein by WB was more difficult in mosquito cells than in BHK cells, requiring significantly longer exposure times. In wtBUNV-infected C6/36 cells NSs protein levels peaked at 24 hpi, and were barely detectable by 76 hpi (Fig. 6D). The NSs4KR protein seemed to peak at the same time during infection, but at lower levels than wt NSs (Fig. 6E). Since mosquito cells have a UPS similar to that in mammalian cells ([42], [43]), the effect of proteasome inhibitors was also tested in these cells. Addition of MG132 to infected cells for 8 h prior to harvest did increase the levels of NSs observed in wtBUNV infected cells (Fig. 6D), confirming that NSs is degraded by the proteasome in mosquito cells. Addition of MG132 seemed to have only very little effect on the levels of NSs4KR, as would be expected.

Taken together, the results showed that the mutations in NSs4KR had no effect on virus growth in different mosquito cell lines, and that the mutant protein did not accumulate to higher levels than the wt protein. The reasons for the levels of NSs4KR remaining significantly below those of wtNSs in mosquito cells remain unclear.

## Discussion

We have demonstrated here that during BUNV infection of mammalian cells the NSs protein is actively targeted for proteasomal degradation. This degradation requires a functional UPS, as well as at least one lysine residue in NSs. Although these findings strongly suggest that NSs is ubiquitylated, this modification could not be demonstrated either *in vitro* or *in vivo*. Failure to demonstrate NSs ubiquitylation *in vitro* may be indicative of temporal regulation of this process during the infection cycle, which may be difficult to replicate in a cell-free lysate. Successful detection of NSs by Western blotting depends on the multiplicity of infection and the time post infection that the samples are taken, as well as the type of host cells supporting the infection. For instance, the levels of viral proteins appeared to be overall lower in A549 cells than in BHK cells. Treating cells with MG132 leads to accumulation of NSs, making it easier to detect, and it would be expected that proteasome inhibition would lead to detection on Western blots of higher molecular-weight species corresponding to ubiquitylated NSs. However, these have not been observed. Possibly the turnover rate of ubiquitylated NSs by de-ubiquitinating enzymes is high, leaving only undetectably low levels of ubiquitylated NSs in the cell lysates. However, adding a de-ubiquitinase inhibitor to infected His_6_-ubiquitin expressing cells did not aid detection of modified NSs in the pull-down assays (not shown). At present, therefore, ubiquitylation of NSs can be neither demonstrated nor excluded.

An alternative explanation for the lack of detectable ubiquitylated NSs might be that it is not NSs itself that is modified, but rather some putative interacting partner. The ubiquitylated partner through its association with NSs could direct NSs for degradation. If this were the case, substitution of the lysine residues in NSs for arginine residues could result in disturbance of the structure of NSs that could weaken or prevent its interaction with the partner, thus rendering NSs resistance to degradation. Future work will examine this possibility.

NSs interacts with Med8, a component of the Mediator complex that regulates RNAPII activity, and this interaction is thought to be involved in the NSs-mediated inhibition of cellular transcription ([35]). Med8 can also be part of an E3 ligase ([34]), which made it a prime candidate for the potential E3 ligase that targets NSs for degradation. However, treatment of infected cells with MLN4924 did not stabilise NSs, indicating that no Cullin-2-based ligases were involved in its degradation and thus ruling out the possible involvement of Med8 as the E3 ligase recognising NSs.

In the experiments described above the inhibitors MG132, epoxomicin and PYR-41 were added at 5–8 hpi, to allow N protein synthesis to commence before treatment. When added from the start of infection, the proteasome inhibitor MG132 and the E1 inhibitor PYR-41 significantly delayed onset of N protein production (by more than 12 h; data not shown). This is an indication that, like many other viruses, BUNV requires an active UPS during the early stages of infection.

Serial passage of wtBUNV, rBUN4KR and rBUNdelNSs2 in BHK cells revealed that both mutant viruses accumulated significantly more non-full-length L segment-derived RNAs, presumed DI RNAs, than the wt virus. This could indicate a subtle role for NSs in regulation of the viral polymerase, perhaps as a quality control mechanism, such that either the lack of NSs or the over-expression of NSs could both result in increased DI production. Experiments are currently underway to test this hypothesis.

Why is NSs degraded during infection of mammalian cells? All the assays that test reported roles of NSs showed the non-degradable NSs4KR mutant protein to function at least as well as the wt NSs protein, if not better. In infected A549 cells, the mutant virus with stabilised NSs had a slight growth advantage over wt virus at 37°C, presumably as a result of increased and prolonged expression of the viral interferon antagonist. This finding demonstrated that the arginine substitutions did not impair the interferon antagonist function of NSs. In interferon-deficient A549-V or BHK cells no difference was observed in growth kinetics between mutant rBUN4KR and wtBUNV. In mice, both viruses were equally virulent, killing the mice in 4–6 days p.i., demonstrating that expressing a stabilised IFN antagonist did not make the virus more virulent. The lack of any negative effect of the 4KR mutations on virus growth in mammalian cells or virulence in mice suggested that perhaps the lysine residues in NSs could be important for a role during infection of the mosquito vector.

In mosquito cells, BUNV infection does not result in shut-off of host cell metabolism, and the NSs protein is expressed at comparatively lower levels than in mammalian cells ([12]). However, production of NSs is required for efficient replication in certain mosquito cell lines and in mosquitoes ([44]). Analysis of virus yields showed no difference in growth between wtBUNV and rBUN4KR in each of three mosquito cell lines, indicating that in these cells the absence of lysine residues in NSs did not impair virus growth.

Contrary to expectation, in *A. albopictus* C6/36 cells NSs4KR was not stabilised, and the protein could only be detected by WB relatively early in infection (24hpi) when cells were infected at high multiplicity. Unlike wt NSs the mutant protein could not be stabilised by treatment with MG132 and therefore degradation of NSs4KR in C6/36 cells would seem to be due to proteases that recognise motifs that are absent in wt NSs. In addition, the levels of NSs4KR expressed during infection were significantly lower than those of wt NSs whereas the levels of N protein were similar, indicating that the levels of mRNA produced by the two viruses were similar. The only difference between the S segment-derived mRNAs is the four nucleotides in the middle of the coding region that change the four lysine residues to arginines in the NSs ORF. A possible explanation for the lower levels of NSs4KR could therefore lie in different rates of synthesis between wt and mutant protein as a result of the relative abundance of the required tRNAs (for lysine codons AAA or AAG compared to arginine codons AGA or AGG). The relative usage of the arginine codons that were introduced into NSs4KR is quite low in mosquito cells (6 and 4 per 1000 versus 11 and 11 per 1000 in BHK cells; (http://www.kazusa.or.jp/codon/cgi-bin/showcodon.cgi?species=7160; http://www.kazusa.or.jp/codon/cgi-bin/showcodon.cgi?species=10036), but is considerably higher in the BUNV genome itself (23 and 9 per 1000). This usage is even higher in the ORF of the NSs4KR mutant (equivalent to 59 and 29 per 1000), which could be a cause of the lower levels of protein synthesis observed. However, because these numbers relate to usage per 1000 codons and as the NSs ORF is only 102 codons long, in absolute numbers translation of the 4KR NSs protein requires only two more of the lower-usage tRNAs than the wt NSs protein. Metabolic labelling followed by immunoprecipitation to investigate the relative rates of synthesis of the two NSs proteins in mosquito cells yielded signals that were too low to get reliable quantification (not shown). Furthermore, due to the overlapping reading frame of the N protein, three out of the four lysine codons of NSs could not be mutated to any other, more abundantly used, arginine codons to investigate this matter further.

In summary, we have generated a mutant virus expressing a stabilized NSs protein with thus far only one observed phenotype: slightly enhanced growth in A549 cells at 37°C. The lysine mutations did not appear to affect the assayable functions of NSs in mammalian cells, or the ability of the mutant virus to replicate in *Ae. albopictus* U4.4 and *Ae. aegypti* Ae cells which have a requirement for NSs expression ([44]). Further studies are in progress to determine whether rBUN4KR shows any phenotype in live mosquitoes.

## Materials and Methods

### Cells and viruses

Vero-E6, BHK, BSR-T7/5 ([45]; kindly provided by K. K. Conzelmann), A549 and A549-NPro ([46]; kindly provided by R. Randall) cells were maintained as described previously ([35], [44], [46]). A549-V cells, stably transformed with the V protein of parainfluenza virus type 5 to block interferon signalling (unpublished), were also provided by R. Randall, and maintained in Dulbecco's minimal essential medium (DMEM) supplemented with 10% fetal calf serum (FCS). A549-6His-Ub cells constitutively expressing 6His-tagged ubiquitin (unpublished) were generously provided by Dr B. Hale, and were maintained in DMEM supplemented with 10% FCS. Mosquito cell lines C6/36 and U4.4 from *Aedes albopictus* and Ae from *Aedes aegypti* were maintained as described by [44]. Stocks of wtBUNV, rBUNdelNSs2, and of the mutant rBUNNSs4KR described here, were prepared as described previously following plaque purification ([35]). The recombinant viruses expressing single-lysine mutants of NSs, rBUNK1, rBUNK2, rBUNK3 and rBUNK4 were not plaque-purified, but working stocks were grown by infecting BHK cells with rescue supernatants.

### Plasmids

Plasmids containing full-length cDNAs of BUNV genome segments or the minigenome under control of T7 promoter and terminator sequences (pT7ribo-BUNL(+), pT7ribo-BUNM(+), pT7ribo-BUNS(+) and pT7ribo-BUNMRen) and pTM-1 derived plasmids expressing NSs, N or L (pTM1-NSs, pTM1-N, pTM1-L) have been described previously ([11], [47]). pTM1-NSs and pT7ribo-BUNS(+) were mutated to change the 4 lysine residues in NSs to arginine residues by Quickchange PCR mutagenesis to create the 4KR versions. The plasmids were amplified using KOD polymerase (Novagen) and primers ivk69 and ivk70. After 18 cycles of PCR amplification the template was destroyed by incubation with DpnI (Fermentas) and the products were transformed into *E. coli* JM109. A similar procedure, using 4KR versions of the plasmids as template, was used to generate the related plasmids expressing NSs-K1 (primers ivk86 and ivk87), -K2 (primers ivk88 and ivk89), -K3 (primers ivk90 and ivk91) and -K4 (primers ivk92 and ivk93) constructs. All primers are listed in Table 1.

**Table 1:** Primers used in this study.

Primer	Name	Aligns to BUNS(+) nts	Sequence 5′–>3′
ivk01	S-Fw mut	1–46*	AGTAGTGTACTCCACCTAAAACTTATGCTATTGTTGAAAATCGCTG
ivk02	S-Rv mut	936–912*	CAGTTCTCGAGCATCAAATACCATTAACAATATAATGTTG
ivk20	NSs-start Fw	104–126*	GGGAAACGTCTCaCATGATGTCGCTGCTAACACCAG
ivk55	3′UTR Rv	892–846*	TCGAGTGTCCCCAACCACCCACCCAAGCAGCTGTTATTTTGTGGGTTGAAAC
ivk69	4KR- Fw	211–278	CTACATTAA**g**GGACGCGAGATTAA**g**ACTAGTCTCGCAAA**g**AGAAGTGAATGGGA**g**GTTACACTTAACC
ivk70	4KR- Rv	273–203	AGTGTAAC**c**TCCCATTCACTTCT**c**TTTGCGAGACTAGT**c**TTAATCTCGCGTCC**c**TTAATGTAGAAGATTCG
ivk86	K1-F	211–237	CTACATTAA**a**GGACGCGAGATTAAGAC
ivk87	K1-R	237–211	GTCTTAATCTCGCGTCC**t**TTAATGTAG
ivk88	K2-F	220–246	GGGACGCGAGATTAA**a**ACTAGTCTCGC
ivk89	K2-R	246–220	GCGAGACTAGT**t**TTAATCTCGCGTCCC
ivk90	K3-F	235–263	GACTAGTCTCGCAAA**a**AGAAGTGAATGGG
ivk91	K3-R	263–235	CCCATTCACTTCT**t**TTTGCGAGACTAGTC
ivk92	K4-F	250–278	GAGAAGTGAATGGGA**a**GTTACACTTAACC
ivk93	K4-R	278–250	GGTTAAGTGTAAC**t**TCCCATTCACTTCTC

* primers with internal mismatch or 5′ tail that did not interfere with PCR amplification.

Mutations introduced by these primers are indicated in bold and lower case.

### Virus rescue

Viruses were rescued as described before ([48]). Briefly, 60 mm dishes of subconfluent BSR-T7/5 cells were transfected, using Lipofectamine-2000, with 1 µg each of pT7ribo-plasmids expressing the three viral RNA segments. The transfected cells were incubated at 33°C until CPE was observed.

### Virus Infection

Viruses were diluted in PBS containing 2% newborn calf serum such that the minimum infection volume (200 µl for a 35-mm dish) contained the required amount of virus for the intended m.o.i. Multi-cycle infections for growth curves were inoculated at m.o.i. 0.01, and single-cycle infections for protein analysis at m.o.i. 5. Cells were infected with virus at 37°C (or 33°C where indicated) for 1 h and the inoculum was replaced with fresh growth medium at t = 1 hpi. For growth curves the supernatants were harvested, clarified by centrifugation at 500×g for 5 min, and stored at −80°C before titration by plaque assay.

Where indicated drugs were used as follows: to block the proteasome the medium on the cells was replaced at 5–6 hpi by medium containing 10 µM MG132 (Calbiochem) or 200 nM Epoxomicin (Sigma), which was left on until harvest. The inhibitor of the E1 ubiquitylation enzyme, PYR-41 (Merck), was used at 50 µM and added at 8 hpi. The NEDD8 E1 activating enzyme inhibitor MLN4924 (kindly provided by Prof. R.T. Hay) was used at 1 µM, and added at 5–6 hpi.

### Plaque assay

The plaque phenotype of viruses and the titres of growth curve and passaging assay samples were determined by plaque assay. Vero-E6 cells were infected with serial dilutions of the samples, and after 1 h incubation at 37°C an overlay of 0.6% agarose and 2% FBS in 1xMEM with was applied. Cells were incubated at 37°C for 5 days and then fixed in 4% formaldehyde for 3 hours before staining with Giemsa stain.

### Biological assay for interferon production

A549 cells in 35 mm dishes were infected with 3 PFU/cell of rBUNdelNSs2 or transfected with 1 µg poly(I∶C) and incubated at either 33°C or 37°C for 24 h. After virus inactivation two-fold serial dilutions of the medium were applied to fresh A549-NPro cells for 24 h. IFN-sensitive encephalomyocarditis virus (EMCV) was then added (0.05 PFU/cell), and cells incubated for 4 days at 37°C. The cells were fixed with formaldehyde and stained with Giemsa stain to monitor development of CPE. The relative IFN units (RIU) are expressed as 2^N^ where N is the number of two-fold dilutions that protect the reporter cells.

### Passaging assay

T25 flasks of BHK cells were infected initially with 5 PFU/cell of wtBUNV, rBUN4KR, or rBUNdelNSs2. The supernatant was harvested when clear signs of CPE were observed. Subsequent passages were performed ‘blind’ (without titrating the viruses): new flasks were infected with a fixed volume of 0.3 ml virus per flask and harvested when CPE was observed or at 3–4 dpi. The supernatants were collected, clarified, and stored at −80°C.

### Minireplicon assay

To measure viral RNA polymerase activity plasmids expressing all the components of an RNP (viral RNA, N protein, L protein) were transfected into subconfluent BSR-T7 cells in quadruplicate reactions in 24-well plates based on the protocol described previously ([11]). Each well was transfected with 80 ng each of TM1-Fluc (internal control firefly luciferase), TM1-BUNN, TM1-BUNL, and 1.6 ng of T7ribo-MRen (expressing the minigenome containing the *Renilla* luciferase ORF in negative orientation between M segment UTRs). Cells were co-transfected with pTM1-derived plasmids (ranging from 1.6 to 160 ng per well) expressing NSs, NSs4KR, or GFP as a negative control. The total amount of DNA transfected was kept constant by the addition of empty TM1 vector. After 24 h of incubation *Renilla* luciferase activity was measured in a luminometer using the Dual Luciferase Assay Kit (Promega), and standardised to the levels of firefly luciferase. Values were normalised to the control reaction where no NSs protein was expressed.

### DNA fragmentation assay

Analysis of DNA fragmentation, as a measure of apoptosis, followed the method detailed by Kohl *et al.* ([9]).

### Western Blotting (WB)

Detection of proteins by Western blotting was performed as described previously ([44]) using the following antibodies: anti-tubulin (1∶5000; clone B512, Sigma), anti-BUNN (1∶5000; ([35])), anti-BUNNSs (1∶500; ([8])), anti-NEDD8 (1∶1000; Epitomics), anti-rabbit IgG-HRP conjugate (1∶5000; Cell Signalling), anti-mouse IgG peroxidase conjugate (1∶5000; Sigma). Typically, exposures for N protein and tubulin were very short (5–15 sec), whereas detection of NSs required much longer exposures ranging from 2 to 30 min.

### Northern Blotting (NB)

For RNA analysis of passaged viruses, the virions were pelleted from 1 ml of supernatant by centrifugation in a benchtop microfuge at 20,000×g for 1.5 h at 4°C. The pellets were resuspended in 140 µl of PBS and the RNA was extracted using the QiAmp viral RNA minikit (Qiagen). The yields of RNA varied considerably but were not directly related to the level of signal of the viral RNA segments. The amount of RNA to be loaded on the gel was determined empirically and ranged from 0.8 to 3.8 μg per lane. The RNA was separated by electrophoresis on a 1.5% agarose/TAE gel, followed by capillary transfer in 10xSSC to a positively charged nylon membrane. Viral genome-sense RNA species of the L-segment were visualised by hybridysation to the DIG-labelled L+ probe and detection by anti-DIG-HRP antibody using the DIG Northern Starter Kit (Roche).

### Virus sequencing

To determine the viral genome sequences after serial passage, the virion RNA was extracted as described above. cDNA to the S segment was synthesised using MMLV Reverse Transcriptase (Promega) with primer ivk01, followed by PCR amplification using KOD polymerase (Novagen) with primers ivk01+ ivk55 and ivk02+ ivk20 (for all primers see Table 1). PCR products were purified from agarose/TAE gels using Promega Wizard kit and their sequence determined commercially (Dundee Sequencing Service).

### Mouse studies

Mouse infections were performed as described previously ([49]). Strain 129/Sv(ev) were purchased from B&K Universal Ltd. Mice were maintained in the Hugh Robson Building Animal Unit, College of Medicine & Veterinary Medicine, University of Edinburgh, UK, in specific-pathogen-free and environmentally enriched conditions with food and water supplied ad libitum. All experimental studies were agreed by the University of Edinburgh Ethical Review Committee and were carried out under the authority of UK Home Office licence 60/4112. Groups of six 4–5 week old mice were anaesthitised and inoculated intracranially with 20 μl PBS with 0.75% BSA (PBSA) containing 1×10^3^ PFU wtBUNV or rBUN4KR virus. The mice were monitored twice daily for up to two weeks and euthanized when displaying 4 or more of the predefined moderate severity signs.
